# Spatial anxiety and self-confidence mediate sex/gender differences in mental rotation

**DOI:** 10.1101/lm.053596.122

**Published:** 2022-09

**Authors:** Linda Arrighi, Markus Hausmann

**Affiliations:** Department of Psychology, Durham University, Durham DH1 3LE, United Kingdom

## Abstract

A recent meta-synthesis study with a sample of >12 million participants revealed that the male advantage in mental rotation (MR) is the largest cognitive sex/gender difference found in psychological literature. MR requires test takers to mentally rotate three-dimensional cubic figures under time restrictions. Previous studies have investigated how biological and social factors contribute to cognitive sex/gender differences in tasks of this type. Spatial anxiety and self-confidence in MR tasks have received less attention. The present study investigated the contribution of these psychological factors to sex/gender differences in MR performance. Participants (*n* = 269) completed two MR tasks that differed in task difficulty. Participants also indicated their self-confidence (for each item) and spatial anxiety. The results revealed that pronounced sex/gender differences in spatial anxiety and self-confidence mediate sex/gender in MR performance, especially when task demands are high. The current findings suggest that task-irrelevant factors that are not spatial cognitive in nature contribute largely to the well-known medium to large sex/gender differences in MR. Future studies should further explore mechanisms underlying cognitive sex/gender differences within a biopsychosocial approach.

Although men's and women's cognitive profiles largely overlap, sex/gender^1^ differences in certain cognitive abilities are consistently reported, albeit with different effect sizes (e.g., Hyde 2005; Torres et al. 2006; Toivainen et al. 2018; Hirnstein et al. 2019). While women tend to outperform men in some verbal tasks, such as speech production (*d* = 0.33) (Hyde 2005), men tend to achieve higher performance in some spatial abilities (Voyer et al. 1995; Reilly and Neumann 2013). Spatial abilities are needed to perceive, localize, visualize, manipulate, and understand relationships between objects in space (Uttal et al. 2013; Newcombe and Shipley 2015).

When compared with other tests of spatial perception (*d* = 0.44) and spatial visualization (*d* = 0.19), mental rotation (MR; *d* = 0.56–0.73) produces the most reliable sex/gender difference (Voyer et al. 1995). In fact, a metasynthesis based on >12 million participants revealed that the male advantage in MR is the largest cognitive sex/gender difference found in the psychological literature (Zell et al. 2015). MR refers to a process in which participants visualize and mentally rotate objects (Voyer et al. 1995). MR is an intrinsic dynamic spatial task in line with a classification by Uttal et al. (2013). In particular, the Mental Rotation Test (MRT) is a well-established psychometric paper–pencil test in which participants are required to mentally rotate three-dimensional (3D) cube figures designed by Shepard and Metzler (1971) and asked to identify which two out of four stimulus figures match a target figure under a time limit (Peters 1995).

Meta-analyses on sex/gender differences in MR have shown medium to large effect sizes in favor of men (Linn and Petersen 1985; Voyer et al. 1995; Voyer 2011; Reilly and Neumann 2013), which have remained relatively stable across the years (Masters and Sanders 1993). Although the male advantage in MR has been shown to be larger in adults compared with children (Voyer et al. 1995), it did not significantly decrease as the year of birth increased. This suggests that the magnitude of sex/gender differences in MR is less affected by the social environment in which participants were raised (Voyer et al. 1995).

Additionally, there are specific task characteristics that affect the size of the sex/gender difference in MR performance (Linn and Petersen 1985; Collins and Kimura 1997; Peters 2005; Voyer 2011). For example, it has been argued that the use of 3D objects might increase sex/gender differences. However, MR tasks involving 2D objects have also shown a male advantage when task difficulty is high (Collins and Kimura 1997). Furthermore, a study by Jansen-Osmann and Heil (2007) did not find sex/gender differences in the speed of mental rotation of 3D cube figures, disconfirming the importance of dimensionality in sex/gender differences. Apart from dimensionality, the size of the rotation angle, the number of rotation axes, and the complexity of the stimuli also contribute to the male advantage (Caissie et al. 2009). Other task factors that might enhance the sex/gender difference in MR are stimulus shape (Amorim et al. 2006; Jansen-Osmann and Heil 2007), stimulus color (Rahe et al. 2022), and response format (e.g., whether the number of correct answers per item is constant) (Hirnstein et al. 2009).

Psychometric MR tasks are usually administered with time constraints. Peters (2005) argued that including a time constraint of any duration makes the task more ecologically valid, as perceptual speed is relevant to spatial abilities in a real-life environment. Time-constrained MR tasks produce larger sex/gender differences than MR tasks administered with no time limits (Peters 2005; Voyer 2011). As sex/gender differences are not eliminated when administered with no time constraints, this suggests that other task-related factors affect the sex/gender difference too (Voyer 2011). However, it is clear that time constraint is one critical factor in MR tasks that will usually amplify the size of the sex/gender difference. Notably, chronometric MR tests, which measure reaction time when identifying whether an object is a rotated or mirrored version of another without a time limit, do not tend to show sex/gender differences (Rahe et al. 2019).

It should be noted that task-related factors can only partially explain sex/gender differences in mental rotation, which are still not fully understood (Halpern and LaMay 2000). A slightly different perspective on how to answer this research question has been offered by studies investigating biological, social, and psychological factors that may affect sex/gender differences in mental rotation performance. Although there is no doubt that biological factors such as sex hormones (Hausmann et al. 2000; Miller and Halpern 2014) and individual differences in structural and functional brain organization (e.g., Hausmann 2017; Hirnstein et al. 2019), social factors such as gender stereotypes (e.g., Halpern et al. 2007; Hausmann 2014), and the interaction between biological and social factors (e.g., Josephs et al. 2003; Wraga et al. 2007; Hausmann et al. 2009) contribute to sex/gender differences in spatial abilities, psychological factors are frequently neglected. This is surprising, as psychological factors have been shown to be particularly good candidates for elucidating interindividual and sex/gender differences in spatial abilities in general and MR in particular.

The current study aimed to replicate the well-known sex/gender difference in MR performance and to investigate to what extent individual differences in psychological factors spatial anxiety and self-confidence contribute to and mediate the effect of sex/gender on MR performance when task demands are high and low. To achieve this, the present study included the more demanding Revised Vandenberg and Kuse Mental Rotations Tests (version MRT-A) (Peters 1995), which involve 2D drawings of 3D cube figures (Shepard and Metzler 1971), and the less demanding Mirror Pictures task—a 2D mental rotation test and subtest of the WILDE-Intelligenz-Test (Jäger and Althoff 1983). Self-confidence was measured on item level of each test. Trait spatial anxiety was measured with a questionnaire after cognitive testing.

Self-confidence (i.e., the certainty that the participant's responses are correct) is known to be generally higher in men than in women, especially in evaluation settings (Lenney 1977). Men's higher self-confidence in their visuospatial performance even occurred when sex/gender differences in spatial performance were not observed (Ariel et al. 2018). However, self-confidence was positively correlated with MR performance (Cooke-Simpson and Voyer 2007). Given that men showed higher self-confidence in MR tasks compared with women, this might partly explain why men on average outperformed women in this study. The sex/gender difference in MR self-confidence was replicated by Estes and Felker (2012), who also found that self-confidence significantly mediated the sex/gender difference in MR performance; that is, more self-confident men revealed higher MR scores than women (Estes and Felker 2012). Furthermore, the positive relationship between self-confidence and MR performance was stronger in men than in women. These studies usually neglected psychological traits that might affect both individuals’ self-confidence and MR performance.

Spatial anxiety is a domain-specific anxiety defined by negative thoughts and feelings when performing spatial tasks (Lawton 1994; Ramirez et al. 2012). A construct similar but not identical to spatial anxiety is self-efficacy, which has been defined as the belief in one's own ability to perform a task (Bandura 1994). Spatial self-efficacy was positively correlated with MR performance in both men and women (Towle et al. 2005). Sex/gender differences in spatial anxiety emerged in children aged 6–12 yr (Lauer et al. 2018) and continued in adulthood (Lawton 1994). Women and girls showed significantly higher spatial anxiety than men and boys (Lawton 1994; Lauer et al. 2018; Alvarez-Vargas et al. 2020). Different aspects of spatial anxiety include navigation anxiety and spatial mental manipulation anxiety (Lyons et al. 2018). Navigation anxiety is defined by negative thoughts when attempting tasks involving directions and wayfinding. Mental manipulation anxiety is an anxiety surrounding spatial visualization, mental rotation, and imagined movement of abstract 3D objects, and hence reflects the demands of MR tasks. Women showed significantly higher navigation and mental manipulation anxiety than men (Lyons et al. 2018). Some evidence of a negative correlation between spatial anxiety and MR performance has been previously shown, with a recent study finding that spatial anxiety and not trait anxiety partially mediated the effect of sex/gender on MR performance; that is, women, higher in spatial anxiety than men, obtained lower performances (Alvarez-Vargas et al. 2020). When looking at within-scale factors identified with exploratory factor analysis, MR and navigation anxiety significantly mediated the effect of sex/gender on MR performance. However, the effect of sex/gender remained significant despite the effects of MR/navigation anxiety. Additionally, a moderate negative correlation between spatial anxiety and MR performance was found in children aged 6–12 yr, suggesting that the detrimental effect of spatial anxiety on MR performance might develop relatively early on (Lauer et al. 2018). Overall, these findings suggest that spatial anxiety is a key factor mediating sex/gender differences in MR. However, the precise mechanism through which spatial anxiety affects MR performance remains unclear.

We hypothesized that men outperform women, especially in the more demanding MRT (hypothesis 1). We also predicted that, on average, women show higher spatial anxiety and lower self-confidence compared with men (hypothesis 2). Critically, it was hypothesized that the sex/gender difference in MR performance are mediated by the sex/gender differences in spatial anxiety and self-confidence, especially when task demands are high (hypothesis 3). Finally, in a series of exploratory analyses, we examined the sex/gender difference in self-confidence at the item level as well as in MR performance at each level of self-confidence (and spatial anxiety).

## Results

### Mental rotation test

**Figure 1.**
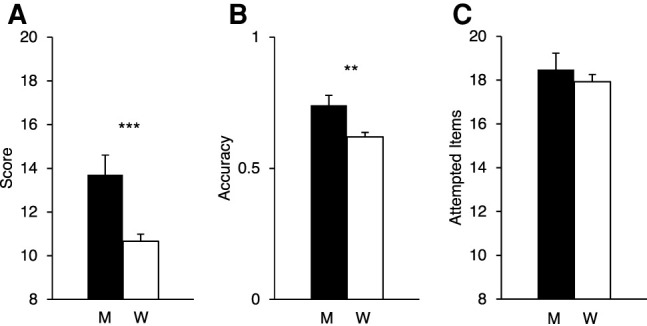
(*A*–*C*) Means (and SEMs) for MRT score (*A*), accuracy (i.e., MRT score divided by number of attempted items) (*B*), and attempted items (*C*). Black bars represent men (M), and white bars represent women (W). (***) *P* < 0.001.

As expected (hypothesis 1), men (13.70 ± 5.70; M ± SD) obtained higher MRT scores compared with women (10.66 ± 4.94; *t*_(267)_ = 3.18, *P* < 0.001, *d* = 0.60). Similarly, the accuracy score (i.e., MRT score divided by number of attempted items) for men (0.74 ± 0.24) was significantly higher than for women (0.62 ± 0.25; *t*_(267)_ = 2.97, *P* = 0.003, *d* = 0.51). The number of attempted items did not differ between men (18.48 ± 4.75) and women (17.93 ± 4.96; *t*_(267)_ = 0.65, *P* = 0.516, *d* = 0.11). Mean and SEMs for MRT scores, accuracy, and number of attempted items are shown in Figure 1, A–C.

### Mirror pictures

**Figure 2.**
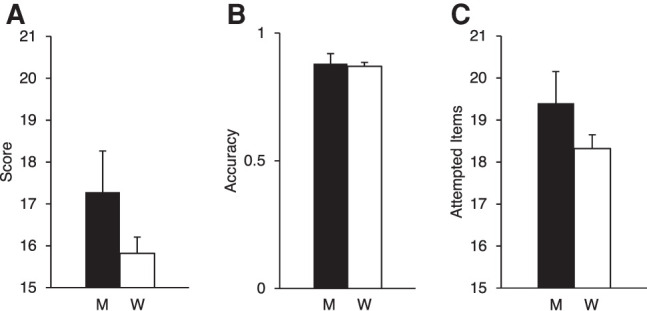
(*A*–*C*) Means (and SEMs) for MP score (*A*), accuracy (*B*), and attempted items (*C*). Black bars represent men (M), and white bars represent women (W).

Men (17.28 ± 6.22) obtained numerically higher MP scores compared with women (15.82 ± 5.88). The sex/gender difference in MP score was not significant (*t*_(267)_ = 1.43, *P* = 0.154*, d* = 0.25). Similarly, the accuracy score (i.e., MP score divided by number of attempted items) did not differ between men (0.88 ± 0.25) and women (0.87 ± 0.23; *t*_(267)_ = 0.34, *P* = 0.733, *d* = 0.06). Finally, the number of attempted items did not differ between men (19.40 ± 4.58) and women (18.32 ± 4.83; *t*_(267)_ = 1.31, *P* = 0.191, *d* = 0.23). Mean and SEMs for MP scores, accuracy, and number of attempted items are shown in Figure 2, A–C.

To compare performances between the two tasks, paired sample *t*-tests and correlations were performed. The results revealed significantly higher test scores in MP than in MRT for men (*t*_(39)_ = −5.06, *P* < .001, *d* = −0.80) and women (*t*_(228)_ = −14.97, *P* < 0.001, *d* = −0.99). The results suggest that despite significant positive correlations for men [*r*(40) = 0.72, *P* < 0.001] and women [*r*(229) = 0.55, *P* < 0.001], the MRT is more demanding than MP for both sexes/genders.

To check for test order effects and interactions with sex, 2 (MRT first, MP first) × 2 (men, women) ANOVAs were carried out on the MRT and MP scores. Neither the main effect nor interaction with “test order” approached significance (all *P*s > 0.237).

### Self-confidence in spatial abilities

For the MRT, as predicted (hypothesis 2), men (5.44 ± 1.07) showed significantly higher self-confidence than women (4.39 ± 1.42; *t*_(66)_ = 5.46, *P* < 0.001, *d* = 0.77). For MP, men (5.82 ± 1.36) showed significantly higher self-confidence than women (5.18 ± 1.45; *t*_(267)_ = 2.59, *P* = 0.01, *d* = 0.44) (see Table 1).

**Table 1.**
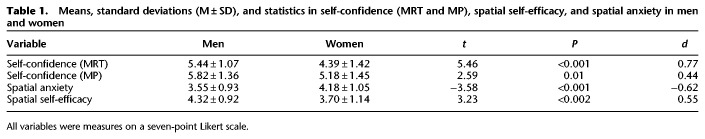
Means, standard deviations (M ± SD), and statistics in self-confidence (MRT and MP), spatial self-efficacy, and spatial anxiety in men and women

To investigate task-specific differences in self-confidence for both sexes/genders, paired *t*-tests were carried out. The results showed higher self-confidence for MP than MRT for men (*t*_(39)_ = −2.442, *P* = 0.019, *d* = −0.39) and women (*t*_(228)_ = −9.824, *P* < .001, *d* = −0.65), suggesting that MP was perceived as less demanding than MRT, which is also supported by participants’ performance in both tasks.

#### Sex/gender differences in self*-*confidence on a trial*-*by*-*trial analysis

To investigate whether sex/gender differences in self-confidence change across test trials, exploratory post-hoc *t*-tests (one-tailed) were performed with sex/gender as between-subject variable and self-confidence as dependent variable. The significance level was set to *P* < 0.01 to correct for multiple comparisons.

**Figure 3.**
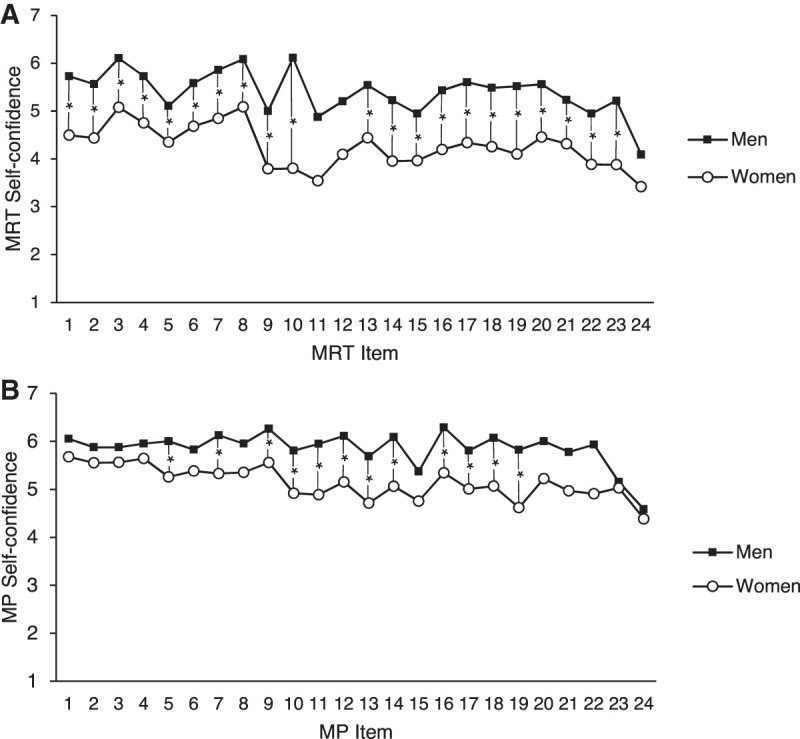
(*A*,*B*) Mean MRT self-confidence (*A*) and MP self-confidence (*B*) in individual items as a function of sex/gender. Self-confidence was measured on a seven-point scale (1 = not at all confident to 7 = extremely confident). (*) *P* < 0.01.

For the MRT, men showed significantly higher self-confidence (*P* < 0.01) for all items (apart from items 11, 12, and 24), with effect size Cohen's *d* ranging between *d* = 0.41 and *d* = 1.34. Please note that the MRT was administered under time restrictions in two blocks: items 1–12 and items 13–24. The results suggest that the sex/gender difference in self-confidence was relatively constant and not susceptible to the specific item and its position (see Fig. 3A).

For MP, the same analysis of men revealed significantly higher self-confidence than women for items 5, 7, 9–14, and 16–19 (all *P*s < 0.01), with effect size Cohen's *d* ranging from *d* = 0.43 to *d* = 0.66. Sex/gender differences in self-confidence for items 1–4 and 20–24, although numerically higher in men than women, were not significant. It is important to note that items in the MP task are organized by incremental difficulty (Lippens 2016), suggesting that sexes/genders only differed in self-confidence for medium task demands. Men and women did not differ in self-confidence in MP for least demanding and most demanding items (see Fig. 3B).

Exploratory post-hoc Mann–Whitney tests were carried out with sex/gender (men and women) as independent variable and the proportions of men and women who got each MP item correct as dependent variables to investigate whether sex/gender differences in performance change across trials in a pattern similar to that of self-confidence. The significance level was set to *P* < 0.01 to correct for multiple comparisons. A significantly higher percentage of men than women got items 2, 10, 11, 13, 17, and 24 correct. This indicates that sex/gender differences in self-confidence in MP do not necessarily correspond to sex/gender differences in performance at the trial level.

### Spatial anxiety (and spatial self-efficacy)

Spatial anxiety and spatial self-efficacy were measured after cognitive testing with Likert scale questionnaires consisting of 12 items each. As predicted, women (4.18 ± 1.05) showed significantly higher spatial anxiety scores than men (3.55 ± 0.93; *t*_(267)_ = −3.58, *P* < 0.001, *d* = −0.62). Similarly, women (3.70 ± 1.14) showed significantly lower spatial self-efficacy scores than men (4.32 ± 0.92; *t*_(267)_ = 3.22, *P* < 0.002, *d* = 0.55) (see Table 1).

### Relationship between psychological variables and spatial abilities

#### Mental rotation test

For the MRT, bivariate correlation analyses were carried out to investigate the relationship between self-confidence and spatial anxiety and the MRT score. Self-confidence during the MRT testing showed a significant positive correlation with MRT score for both men (*r*_(40)_ = 0.61, *P* < 0.001) and women (*r*_(229)_ = 0.43, *P* < 0.001). Direct comparison (Fisher's *r*-to-*z* transformation) revealed no significant difference (*z* = 1.41, *P* > 0.05), indicating higher MRT scores in more confident men and women.

Spatial anxiety showed a significant negative correlation with the MRT score for men (*r*_(40)_ = −0.51, *P* < 0.001) and women (*r*_(229)_ = −0.47, *P* < 0.001), and no significant difference between men and women (*z* = 0.30, *P* > 0.05), indicating higher MRT scores in less spatially anxious men and women.

**Figure 4.**
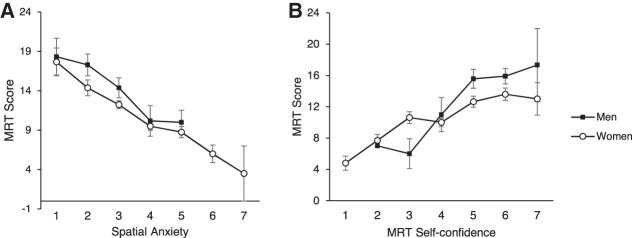
(*A*,*B*) Mean MRT score as a function of sex/gender at different MRT self-confidence ratings (1–7) (*A*) and spatial anxiety ratings (1–7) (*B*). Error bars indicate 1 SE. Black dots represent men, and white dots represent women. The missing data points indicate no man in the corresponding category.

To further dissect the relationship between MRT self-confidence/spatial anxiety and MRT score, participants were divided into seven groups based on average self-confidence/spatial anxiety scores, and average MRT scores were calculated for each group (see Fig. 4A,B). All men showed average self-confidence score >2. Exploratory post-hoc *t*-tests (uncorrected for multiple testing) were carried out to investigate sex/gender differences in MRT score at different self-confidence scores. For an average self-confidence score of 5, men (*N* = 14, 15.57 ± 4.50) showed a higher MRT score compared with women (*N* = 45, 12.64 ± 4.79; *t*_(57)_ = 1.99, *P* = 0.048, *d* = 0.62). For all other average self-confidence scores, men and women showed no differences in MRT scores (all *P*s > 0.082). Similarly, all men showed average spatial anxiety <6. Post-hoc *t*-tests (uncorrected for multiple testing) were carried out to investigate sex/gender differences in MRT score at different spatial anxiety scores. For all average spatial anxiety scores, post-hoc comparisons revealed no sex/gender differences in MRT scores (all *P*s > 0.083).

#### Mirror pictures

The bivariate correlation between MP self-confidence and the MP score was significant for men (*r*_(40)_ = 0.80, *p* < 0.001) and women (*r*_(229)_ = 0.50, *P* < 0.001), albeit stronger for men than women (*z* = 3.02, *P* < 0.05).

Spatial anxiety showed a significant negative correlation with MP score for men (*r*_(40)_ = −0.52, *P* < 0.001) and women (*r*_(229)_ = −0.42, *P* < 0.001) and did not differ between the sexes/genders (*z* = 0.55, *P* > 0.05), indicating higher MP scores in less spatially anxious men and women.

**Figure 5.**
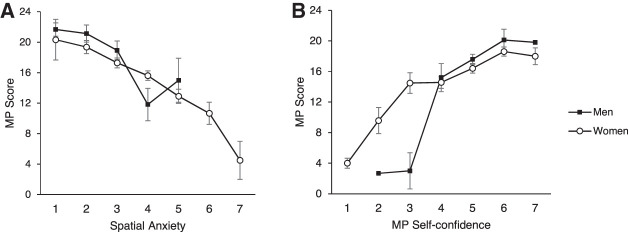
(*A*,*B*) Mean MP score as a function of sex/gender at different MP self-confidence ratings (1–7) (*A*) and spatial anxiety ratings (1–7) (*B*). Error bars indicate 1 SE. Black dots represent men, and white dots represent women. The missing data points indicate no man in the corresponding category.

To further dissect the relationship between MP self-confidence/spatial anxiety and MP score, participants were divided into seven groups based on average self-confidence/spatial anxiety. Average MP scores were calculated for each group (see Fig. 5A,B). All men showed average self-confidence score >2. Exploratory post-hoc *t*-tests (uncorrected for multiple testing) were carried out to investigate sex/gender differences in MP score at different self-confidence scores. No significant differences in MP performance between men and women were shown (all *P*s > 0.096). Similarly, all men showed average spatial anxiety <6. Post-hoc *t*-tests (uncorrected for multiple testing) were carried out to investigate sex/gender differences in MP score at different spatial anxiety scores. Women with average spatial anxiety of 4 (*N* = 78, 15.62 ± 5.55) obtained a higher MP score compared with equally spatially anxious men (*N* = 11, 11.82 ± 7.05; *t*_(87)_ = −2.05, *P* = 0.043, *d* = −0.66). In all other groups, men and women showed no differences in MP scores (all *P*s > 0.243).

### Mediation analysis

**Figure 6.**
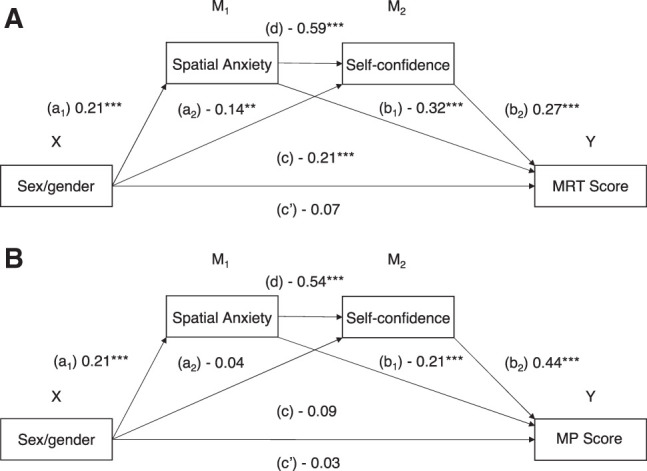
(*A*,*B*) Double-mediation model in path diagram form corresponding to a model with a dichotomous independent variable with two categories (men and women) and with two mediators, spatial anxiety (M_1_) and self-confidence (M_2_), through which sex/gender (X) exerts its effect on MRT score (Y) (*A*) and MP score (Y) (*B*). Of interest are the indirect effects of X and the direct effect, quantified as c′. The total effect of X on Y, denoted by c, is the sum of X's direct and indirect effects on Y. *N* = 269. (**) *P* < 0.01, (***) *P* < 0.001.

Two double-mediation analyses were performed, one for MRT scores and one for MP scores, using model 6 from the PROCESS macro for SPSS (Hayes 2022) to further investigate whether sex/gender predicted spatial scores directly or whether the effect was mediated through self-confidence and spatial anxiety. The hypothetical mediation model included sex/gender as a dichotomous independent variable (men coded as 1 and women coded as 2), spatial anxiety and self-confidence as mediators, and MRT/MP scores as dependent variable. Bias-corrected bootstrap confidence intervals (95%) for interference about indirect effects were used with 5000 samples (generated by stratifying the resampling in each group). The total effect model (*c* path) and the mediation model (*c*′ path) with regression coefficient β are shown for MRT in Figure 6A and for MP in Figure 6B.

#### Mental rotation test

The mediation analysis revealed a significant total effect (*c* path) of sex/gender on MRT score (*R*^*2*^ = 0.04, *F*_(1,267)_ = 12.35, *P* < 0.001). The two mediators added to this prediction accounted for the additional 25.7% of explained variance (*R*^*2*^ = 0.30, Δ*R*^*2*^ = 0.257, *F*_(3,265)_ = 38.00, *P* < 0.001). The association between participants’ sex/gender and spatial anxiety (path *a*_*1*_ = 0.63, SE = 0.18, *t*_(268)_ = 3.58, *P* < 0.001, 95% CI [0.28, 0.98]) was positively and statistically significant, indicating that spatial anxiety was higher in women (coded as 2). The association between participants’ sex/gender and self-confidence (path *a*_*2*_ = −0.55, SE = 0.19, *t*_(267)_ = −2.85, *P* = 0.005, 95% CI [−0.93, −0.17]) was negatively and statistically significant, indicating that self-confidence was lower in women. The association between spatial anxiety and self-confidence (path *d* = −0.80, SE = 0.07, *t*_(267)_ = −12.24, *P* < 0.001, 95% CI [−0.93, −0.67]) was negatively and statistically significant. The influence of spatial anxiety on MRT score (path *b*_*1*_ = −1.55, SE = 0.32, *t*_(266)_ = −4.81, *P* < 0.001, 95% CI [−2.18, −0.92]) and that of self-confidence on MRT score (path *b*_*2*_ = 0.97, SE = 0.24, *t*_(266)_ = 4.00, *P* < 0.001, 95% CI [0.49, 1.45]) were both significant, indicating that participants high in spatial anxiety and low in self-confidence obtained lower MRT scores. The total indirect effect was significant (path *ab* = −2.00, SE = 0.43, 95% CI [−2.87, −1.17]). After adding the mediators in the model, the influence of sex/gender on MRT score became not significant (path *c*′ = −1.04, SE = 0.77, *t*_(266)_ = −1.35, *P* = 0.179, 95% CI [−2.56, 0.48]), but *c*′ path was not equal to 0, indicating a partial mediation. The results indicate that spatial anxiety and self-confidence mediate the association between sex/gender and MRT score.

#### Mirror pictures

The mediation analysis revealed the total effect (*c* path) of sex/gender on MRT score (*R*^*2*^ < 0.01, *F*_(1,267)_ = 2.05, *P* = 0.154) was not significant. The two mediators added to the initial prediction accounted for the additional 32.2% of explained variance (*R*^*2*^ = 0.33, Δ*R*^*2*^ = 0.322, *F*_(3,265)_ = 44.09, *P* < 0.001). The indirect effect of sex/gender on MP score through both spatial anxiety and self-confidence was significant (*ab* = −0.85, SE = 0.27, 95% CI [−1.43, −0.37]). Although the total effect (*c* path) between sex/gender and MP score was not significant, the significant indirect effect suggests that spatial anxiety and self-confidence mediate the association between sex/gender and performance even in the less sex/gender-sensitive MP. As the *c*′ path (−0.44) was not equal to 0, this is a case of partial mediation.

## Discussion

The present study replicated the well-known sex/gender differences in MRT (hypothesis 1). Men outperformed women in the MRT task with a medium effect size (*d* = 0.60), which is in line with several meta-analyses (*d* = 0.56–0.73) (Linn and Petersen 1985; Voyer et al. 1995; Voyer 2011; Reilly and Neumann 2013). The male advantage in MRT was independent of the number of attempted items, as has been suggested previously (Hirnstein et al. 2009; Estes and Felker 2012). In the less demanding MP task, men's performance was numerically higher than women's, but this performance difference was not significant, which is in line with previous findings in studies using comparable tasks (Collins and Kimura 1997). As predicted, self-confidence was positively correlated with MRT (*r* = 0.49) and MP (*r* = 0.55) performance, which is similar in size to previous studies using a comparable self-confidence protocol (*r* = 0.56) (Estes and Felker 2012). Although the strength of the relationship between self-confidence in MRT and MRT performance did not differ between men and women, men's self-confidence in MRT/MP is significantly higher than women's, especially for the MRT. In line with previous studies (Cooke-Simpson and Voyer 2007; Estes and Felker 2012), the findings suggest that self-confidence in MRT/MP is (1) a powerful predictor of MRT/MP performance and (2) partly accounts for the differences in MR performance between and within sex/gender groups, especially when task demands are high. However, although men showed higher self-confidence in MP than women, men and women did not significantly differ in MP performance, suggesting either no direct causal relationship between self-confidence and MR performance exists or that task characteristics played a significant role.

When looking at self-confidence on a trial-by-trial basis, further differences between the two MR tasks emerged. For MRT, most items showed a significant sex/gender difference in self-confidence favoring men, regardless of specific item characteristics or order position. Some of the items showed only numerically higher self-confidence in men (11, 12, and 24), which may be attributable to the fact that only a minority of participants completed those items under time restrictions, as they were at the end of each set. The effect size of the sex/gender difference in self-confidence was particularly large for item 10. Item 10 was one of five MRT items whose figures have occluded parts—a characteristic that was found to increase the size of the sex/gender difference in MRT performance (Voyer and Hou 2006). Self-confidence in MP showed a slightly different pattern, not only because the MP task was overall less demanding but also because items in this task were arranged in an order of incremental difficulty. Although self-confidence showed a significant sex/gender difference for items of medium difficulty, men and women showed no differences in self-confidence for easier and more difficult MP items. The different results for MRT and MP suggest that task difficulty is an important factor that contributes to sex/gender differences in both self-confidence and performance. It is plausible that the medium/high range of difficulty characterizing all MRT items is one critical factor that makes the MRT the most sex/gender-sensitive task in the psychological literature (Zell et al. 2015).

Self-confidence in MRT/MP and the certainty that the participant's responses are correct in those tasks depends not only on specific task characteristics but also on individuals’ psychological traits such as spatial anxiety (a domain-specific anxiety defined by negative thoughts and feelings when performing spatial tasks) (Lawton 1994; Ramirez et al. 2012) and self-efficacy (a related construct that has been defined as the belief in one's own ability to perform a task) (Bandura 1994). In the present study, we focused on spatial anxiety, which was negatively correlated with MRT (*r* = −0.50) and MP (*r* = −0.44) performance. The correlation coefficients in the present study were larger than were previously found (*r* = −0.20) (Alvarez-Vargas et al. 2020). The strong correlation found in the present study may be attributed to the specific statements used in the spatial anxiety scale, which referred to activities that involve visualization and mentally rotating abstract objects. Also, Alvarez-Vargas et al. (2020) did not set time constraints. Previous studies found similar effects of test/math anxiety under time restrictions. For example, high test anxiety participants obtained a lower score in the timed compared with the nontimed condition, which was not observed in the low test anxiety participants (Kellogg et al. 1999; Orfus 2008). Therefore, it is plausible that spatial anxiety also became more relevant in the timed MR tasks of the present study and might explain why high spatial anxiety participants obtained lower MR scores. Finally, the correlations between spatial anxiety and MRT/MP performance were similar for men and women in the present study, suggesting spatial anxiety contributed to individual differences in both MR tasks between and within sex/gender groups (Estes and Felker 2012). Very similar findings were revealed for spatial self-efficacy. In line with previous studies (e.g., Towle et al. 2005; Miola et al. 2021), the present study found a significant positive relationship between spatial self-efficacy and MR performance in both men and women, and spatial self-efficacy as a significant mediator of sex/gender and MR performance. Due to page count restrictions and the conceptual overlap with spatial anxiety, the findings are available in the Supplemental Material, Appendix A.

The present study suggests that self-confidence and spatial anxiety are important psychological factors that partially mediate the effect of sex/gender on performance in both MR tasks. For the MRT, the current findings add on to previous studies that found significant partial mediation of self-confidence and spatial anxiety on the association between sex/gender and MR performance (Estes and Felker 2012; Alvarez-Vargas et al. 2020). However, both factors have never been combined in a single mediation model, and task difficulty has not been considered in this context. For MP, the present study found that self-confidence and spatial anxiety mediated the association between sex/gender and MR performance in a less demanding and less sex/gender-sensitive 2D task, which adds to the body of evidence highlighting the importance of psychological variables as mediators of cognitive performance (Towle et al. 2005; Cooke-Simpson and Voyer 2007; Estes and Felker 2012; Sutin et al. 2019; Alvarez-Vargas et al. 2020). One important question that emerges from the findings of the current study is that of causation. The current study has investigated the significance and direction of the correlation between sex/gender, spatial anxiety (and self-efficacy), self-confidence, and MR performance. However, direct causal relationships between the investigated psychological factors and MR performance have not yet been demonstrated. The significant findings of this study, and specifically the mediation models, highlight that the causality between psychological factors and MR performance is worth investigating further through experimental studies. While Estes and Felker (2012) found some evidence of a causal relationship between self-confidence and MR performance, spatial anxiety (and spatial self-efficacy) have never been experimentally manipulated in the context of MR tasks.

Additionally, the findings of the mediation analyses align with the cognitive appraisal model (Smith and Ellsworth 1987). According to the cognitive appraisal theory, both psychological traits and situational factors influence whether individuals appraise a testing situation as a challenge or threat (Wiese-Bjornstal et al. 1998; Li 2009; Tomaka and Magoc 2021). Spatial anxiety, similarly to cognitive test anxiety, may be related to lower self-confidence and lower test performance by an effect on emotional/physiological arousal during cognitive tasks, task-irrelevant thoughts, and attention (Bargh and Cohen 1978; Seibert and Ellis 1991; Cassady and Johnson 2002; Sullivan 2002; Roos et al. 2021). It is plausible that individuals high in spatial anxiety experienced negative cognitive appraisal, increased arousal, and, in turn, task-irrelevant thoughts while completing the MRT. All of these factors, when considered together, could offer an explanation for the propensity for lower self-confidence and lower performance by participants high in spatial anxiety.

The unequal size of the two sex/gender groups in the current online study is an important limitation that might potentially limit the generalization of the findings. However, it is important to note that MRT and MP scores in men and women were very similar to previous studies (Hausmann et al. 2009; Peters 1995). Another online Qualtrics study found almost identical MRT scores (men: 13.70 ± 5.70 vs. 14.24 ± 7.35, women: 10.66 ± 4.94 vs. 10.45 ± 6.47) (Alvarez-Vargas et al. 2020). Also, the effect size of the sex/gender difference in MRT self-confidence scores (*d* = 0.77) is of a similar large size to that in previous studies (*d* = 1.04) (Estes and Felker 2012). Therefore, we are inclined to believe that the current sample was fairly representational despite differences in group sizes.

In addition, future studies might want to revisit the proposed link between task difficulty and sex/gender differences in self-confidence in MR tasks by manipulating task difficulty more thoroughly and potentially on a trial-by-trial basis with and without time constraints, which typically increase the male advantage in MR tasks (Voyer 2011). Also, it might be interesting to investigate whether the observed differences reported here are sex/gender-specific or also apply to other groups who differ in spatial anxiety and self-confidence, such as people from different ethnicities, education levels, income ranges, etc. (Rushton and Jensen 2005; Zahodne et al. 2017). Future studies might also want to investigate physiological correlates of individual differences in spatial anxiety, self-confidence, and spatial self-efficacy when performing MR tasks, building their theoretical models in line with the findings of the current study. Finally, similar studies should be conducted with a more diverse, less binary concept of sex and gender. In fact, there are hardly any studies that operationalized nonbinary measures of sex/gender (Cameron and Stinson 2019) that also take into account the potential mismatch between a participants’ sex assigned at birth versus their gender identity. A way to encompass the diversity of sexes/genders is to add cisgender and noncisgender categories alongside the binary ones (see the Supplemental Material, Appendix B; Chan 2019).

In sum, the present study replicated direction and size of the sex/gender differences previously reported in MR studies. We also identified self-confidence and spatial anxiety as two major psychological factors that mediate the effect of sex/gender on MR, which is known to be the most sex/gender-sensitive cognitive ability (Zell et al. 2015). The results suggest that the well-known sex/gender difference in MR (with men outperforming women) primarily occurs when men and women differ in spatial anxiety (and spatial self-efficacy), which leads to reduced self-confidence while performing the task, regardless of task difficulty.

## Materials and Methods

### Participants

Participants were recruited through volunteer sampling by advertising on a participant pool platform and an email newsletter service. All participants entered a free prize draw to win one of two £10 Amazon vouchers as incentive to take part in the study. In addition, Psychology students received course credits. Based on previous effect sizes found by studies testing sex/gender differences in mental rotation, a power analysis recommended a sample size of 45 participants in each group to detect an effect size Cohen's *d* = 0.60, with α = 0.05 and β = 0.80. Due to the COVID-19 pandemic, the experiment was moved online, and sample size was increased where possible to account for potential noise due to the online data collection. Participants were naïve to the study hypotheses and the sex/gender-sensitive nature of the study and had not participated in other mental rotation tasks. The present study was approved by the local Ethics Subcommittee at Durham University. All participants completed a consent form prior to participation.

In total, data were collected from 363 participants. Data from 94 participants were excluded because data were incomplete. Data from 269 participants were included in the analysis: 40 men (i.e., males assigned at birth) and 229 women (i.e., females assigned at birth). The participants’ sex was determined by asking “What was the sex you were assigned at birth?” Participants’ age did not significantly differ between men (20.18 ± 3.75, M ± SD) and women (19.91 ± 3.28; *t*_(267)_ = 0.456, *P* = 0.649, *d* = 0.08). The majority of participants were first year undergraduate Psychology students (*n* = 239) at Durham University. Participants of the sample identified as White (70.6%), Asian (24.9%), Black (1.1%), Japanese (0.4%), and other (3.0%).

### Design

The study was carried out on the online platform Qualtrics. The study was self-paced apart from the two MR tasks, which included self-confidence questions. After testing, participants completed a spatial anxiety scale and a spatial self-efficacy questionnaire. Participants were then asked demographic questions and debriefed. Participants on average completed the experiment in 2 h, with cognitive testing lasting no longer than 20 min in total.

### Materials

#### Mental rotation tests

The MRT-A version of the Revised Vandenberg and Kuse Mental Rotations Tests (MRTs) (Peters 1995) is a very established test (>1000 citations). The test contains two sets of 12 items. Each item consists of five Shepard and Metzler (1971) cube figures. A target ﬁgure is located on the left, and four stimulus ﬁgures are on the right. Two of these ﬁgures are rotated versions of the target ﬁgure. The other two ﬁgures cannot be rotated to match the target ﬁgure, as they show different objects altogether. If both matching ﬁgures are correctly identiﬁed, one point is given. Thus, the maximum score in this test is 24 points. A higher score in this test indicates better MR performance.

The mirror pictures (MPs) is a subtest of the WILDE-Intelligenz-Test (Jäger and Althoff 1983). The test contains 24 items of increasing difficulty (Lippens 2016), and each item consists of five simple line drawings. Four drawings show an identical ﬁgure but rotated. One of the drawings is a mirrored/flipped figure and therefore cannot be rotated to match the other four drawings. If the mirror ﬁgure is identiﬁed correctly, one point is given, resulting in a maximum score of 24 points. A higher score in this test indicates better MR performance.

Both MR tests have shown sex/gender differences but with different effect sizes. While Hausmann et al. (2009) revealed the well-known medium effect size for the MRT (*d* = 0.69), the same study revealed that the sex/gender difference in MP was only small (*d* = 0.34). To avoid test order effects, both mental rotation tests were administered to participants in a randomized order.

In line with Estes and Felker (2012), participants rated their self-confidence after each item of the two spatial tests. Specifically, participants were asked “How confident are you that you answered the previous question correctly?” The responses were scored on a seven-point Likert scale (1 = not at all confident to 7 = extremely confident). Only self-confidence scores of attempted items were included in the analyses.

For the MRT, participants were allowed 4 min for each set of 12 items (8 min in total). For the MP, participants were given 5 min for one set of 24 mirrored ﬁgures.

#### Spatial anxiety

To investigate the extent to which spatial anxiety can explain sex/gender differences in cognitive performance, a spatial anxiety scale was adapted from the math anxiety scale (Zakariya 2018).

The spatial anxiety scale consisted of 12 statements (see Table 2). Participants were asked to indicate on a seven-point Likert scale (1 = do not identify at all to 7 = strongly identify) how much they identified with the statements. The average score was calculated after appropriate reverse coding. This resulted in a spatial anxiety score ranging from 1 to 7, with a mean score of 4 indicating average spatial anxiety (4 = indifferent). To avoid any priming effects on test performance, the spatial anxiety scale was administered directly after cognitive testing was completed. The internal consistency of the spatial anxiety scale was high (Cronbach's α = 0.87).

**Table 2.**
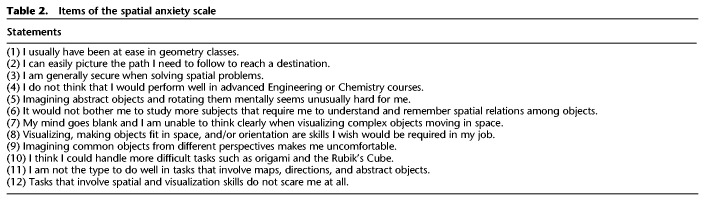
Items of the spatial anxiety scale

The original test battery also included a questionnaire on spatial self-efficacy (M Hausmann, unpubl.). Participants were asked to indicate on a seven-point Likert scale (1 = not at all confident to 7 = extremely confident) how confident they were in their ability regarding 12 statements related to spatial navigation, spatial imagery, and MR tasks. The internal consistency of the spatial self-efficacy scale was high (Cronbach's α = 0.90). Due to the high correlation with the spatial anxiety score and to avoid multicollinearity issues (i.e., a strong negative correlation between spatial self-efficacy and spatial anxiety; *r*_(269)_ = −0.756, *P* < 0.001), the present study reported self-efficacy results only briefly. A more detailed report of the self-efficacy results is in the Supplemental Material, Appendix A.

## Supplementary Material

Supplemental Material

## Footnotes

1 The terms sex and gender are often used interchangeably in the academic literature, although they refer to different concepts. Sex is primarily used to categorize individuals as either males or females, based on the characteristics of their reproductive system, whereas gender is typically used to refer to social factors related to an individual's sex or to the individual's gender identity. In line with a number of recent suggestions (see Hausmann 2021), we use the term sex/gender throughout, since it is not always clear whether results to date are related to sex, gender, or both.
