# Supplementary material for: Spatial anxiety and self-confidence mediate sex/gender differences in mental rotation
Source: Learn Mem. 2022 Sep;29(9):312–20. doi: 10.1101/lm.053596.122 (PMC9488019; doi:10.1101/lm.053596.122)
Supplement: Supplemental Material [file supp_29_9_312__DC1.html]

Supplemental Material 

# Spatial anxiety and self-confidence mediate sex/gender differences in mental rotation

## Supplemental Material

- Supplemental\_Material.docx
