# Supplementary material for: Spatial anxiety and self-confidence mediate sex/gender differences in mental rotation
Source: Learn Mem. 2022 Sep;29(9):312–20. doi: 10.1101/lm.053596.122 (PMC9488019; doi:10.1101/lm.053596.122)
Supplement: Supplemental Material [file supp_29.9.312_Supplemental_Material.docx]

**Appendices**

**Appendix A: Spatial Self−efficacy Analyses**

**A.1 How is spatial self−efficacy correlated with MRT and MP score in men and women?**

Bivariate correlation analyses were carried out to investigate how spatial self−efficacy relates to MRT and MP score. Spatial self−efficacy showed a significant positive correlation with the MRT score, *r*(269)=.36, *p*<.001, and MP score, *r*(269)=.34, *p*<.001. The correlation coefficients for men (MRT *r*(40)=.30, *p*=.063, MP *r*(40)=.41, *p*=.009) and women (MRT *r*(229)=.35, *p*<.001, MP *r*(229)=.32, *p*<.001) were normalised with the Fisher’s *r* to *z*−transformation and compared. The comparison did not show a significant difference between men and women for MRT or MP (*z*<0.51, *p*>.05), indicating that participants higher in spatial self−efficacy obtained higher scores in both tasks and that the effect was similar in men and women.

To further dissect the relationship between spatial self−efficacy and MRT and MP score, participants were divided in 7 groups based on average spatial self−efficacy. Average MRT and MP scores were calculated for each group. All men showed average spatial self−efficacy > 2 and < 7. Post−hoc comparisons were carried out to investigate sex/gender differences in MRT/MP score at different spatial self−efficacy scores. Men with an average spatial self−efficacy of 5 (N=10, 17.20 ± 4.21) obtained a higher MRT score compared to women with equal spatial self−efficacy (N=26, 13.04 ± 3.82), *t*(34)=2.85 *p*=.007, *d*=.06. In all other groups (containing a number of men <14), men and women showed no differences in MRT or MP scores, all *p*s>.102.

The findings suggest that participants relatively accurately estimated their MRT performance regardless of their spatial self−efficacy ratings. Men and women of equal spatial self−efficacy mostly performed equally.

**A.2 Is the association between sex/gender and MRT/MP score mediated by spatial self−efficacy?**

Preliminary regression models identified self−efficacy as a significant predictor of MRT score in women, *R^2^*=.12, *F*(1,227)=30.83, *p*<.001. Furthermore, self−efficacy was a significant predictor of MP scores in men, *R^2^*=.17, *F*(1,38)=7.69, *p*=.009, and women, *R^2^*=.10, *F*(1,227)=25.37, *p*<.001. Double mediation analyses were performed to further investigate whether sex/gender affected MRT / MP score directly or whether the effect was mediated through self−efficacy and self−confidence. These mediation analyses are similar to those in the main manuscript with spatial self−efficacy as mediator 1 instead of spatial anxiety. The analyses revealed that the indirect effect of sex/gender on the MRT score through spatial self−efficacy (path *a_1_b*=−0.40, SE=.20, 95% CI [−0.85, −0.06]) and the indirect effect of sex/gender on the MP score through spatial self−efficacy (path *a_1_b*=−0.26, SE=.01, 95% CI [−0.06, −0.003]) were significant.

**Appendix B: Gender Analysis**

Previous studies in the context of MR performance have hardly taken both sex and gender into account and the added diversity that comes with a sex/gender variable. The main analyses in this manuscript focused on the binary variable referred to as sex/gender. Additionally, a non−binary variable taking into account both sex at birth and gender identity was created to look at how this affected the results. The 3 resulting groups were: cisgender women (N=219), cisgender men (N=40), and non−cisgender women (N=10).

All significant effects remained when running all the analyses excluding non−cisgender participants. However, the authors feel that eliminating non−cisgender is a reductive approach. When including non−cisgender women as additional category, the MRT performance of non−cisgender women (12.20 ± 5.22) was numerically in between that of the participants assigned to the binary cisgender categories (cisgender women 10.58 ± 4.93, cisgender men 13.70 ± 5.70) and not significantly different from that of cisgender men (*t*(48)=0.76, *p*=.454, *d*=.27) and cisgender women (*t*(227)=−1.01, *p*=.313, *d*=−.33). Similarly, the MP performance of non−cisgender women (16.40 ± 7.52) was numerically in between that of the participants assigned to the binary cisgender categories (cisgender women 15.79 ± 5.82, cisgender men 17.27 ± 6.22) and not significantly different from that of cisgender men (*t*(48)=0.38, *p*=.704, *d*=.14) and cisgender women (*t*(227)=−0.32, *p*=.751, *d*=−.10).

In conclusion, there are differences in performance when looking at non−binary gender categories, however the effect sizes in this case are much smaller than when comparing binary categorisations of sex/gender. Future studies should aim to include much larger numbers of non−cisgender participants to better examine the effects of the combination of sex and gender on MR performance.
